# Identification of ferredoxin II as a major calcium binding protein in the nitrogen-fixing symbiotic bacterium *Mesorhizobium loti*

**DOI:** 10.1186/s12866-015-0352-5

**Published:** 2015-02-04

**Authors:** Roberto Moscatiello, Mattia Zaccarin, Flavia Ercolin, Ernesto Damiani, Andrea Squartini, Antonella Roveri, Lorella Navazio

**Affiliations:** Department of Biology, University of Padova, Via U. Bassi 58/B, 35131 Padova, Italy; Department of Molecular Medicine, University of Padova, Viale G. Colombo 3, 35131 Padova, Italy; Department of Biomedical Sciences, University of Padova, Viale G. Colombo 3, 35131 Padova, Italy; Department of Agronomy, Food, Natural Resources, Animals and Environment, DAFNAE, University of Padova, Viale dell’Università 16, 35020 Legnaro Padova, Italy

**Keywords:** Calcium binding proteins, Calcium homeostasis, Ferredoxin II, *Mesorhizobium loti*, Nitrogen fixation, Rhizobium-legume symbiosis

## Abstract

**Background:**

Legumes establish with rhizobial bacteria a nitrogen-fixing symbiosis which is of the utmost importance for both plant nutrition and a sustainable agriculture. Calcium is known to act as a key intracellular messenger in the perception of symbiotic signals by both the host plant and the microbial partner. Regulation of intracellular free Ca^2+^ concentration, which is a fundamental prerequisite for any Ca^2+^-based signalling system, is accomplished by complex mechanisms including Ca^2+^ binding proteins acting as Ca^2+^ buffers. In this work we investigated the occurrence of Ca^2+^ binding proteins in *Mesorhizobium loti*, the specific symbiotic partner of the model legume *Lotus japonicus*.

**Results:**

A soluble, low molecular weight protein was found to share several biochemical features with the eukaryotic Ca^2+^-binding proteins calsequestrin and calreticulin, such as Stains-all blue staining on SDS-PAGE, an acidic isoelectric point and a Ca^2+^-dependent shift of electrophoretic mobility. The protein was purified to homogeneity by an ammonium sulfate precipitation procedure followed by anion-exchange chromatography on DEAE-Cellulose and electroendosmotic preparative electrophoresis. The Ca^2+^ binding ability of the *M. loti* protein was demonstrated by ^45^Ca^2+^-overlay assays. ESI-Q-TOF MS/MS analyses of the peptides generated after digestion with either trypsin or endoproteinase AspN identified the rhizobial protein as ferredoxin II and confirmed the presence of Ca^2+^ adducts.

**Conclusions:**

The present data indicate that ferredoxin II is a major Ca^2+^ binding protein in *M. loti* that may participate in Ca^2+^ homeostasis and suggest an evolutionarily ancient origin for protein-based Ca^2+^ regulatory systems.

**Electronic supplementary material:**

The online version of this article (doi:10.1186/s12866-015-0352-5) contains supplementary material, which is available to authorized users.

## Background

Rhizobia are Gram-negative soil bacteria, which establish in the rhizosphere an intimate mutualistic interaction with leguminous plants. During this symbiosis, in which new organs called nodules are *de novo* generated on plant roots, the microsymbiont provides the plant with fixed nitrogen, receiving in turn fixed carbon from the host [1]. Nitrogen-fixing symbiosis is one of the most important beneficial plant-microbe interactions, supplying dozens of million tons of reduced nitrogen every year to agricultural systems [2]. The rhizobial bacterium *Mesorhizobium loti* and the leguminous plant *Lotus japonicus*, whose genomes have been sequenced [3,4], are widely used as model organisms in the study of rhizobium-legume symbiosis [5]. In the last decade a great deal of scientific attention has been focused on the biochemical dialogue that is progressively established in the rhizosphere, based on the reciprocal exchange of diffusible signals between the two symbiotic partners in the soil. In particular, the role of calcium as critical intracellular messenger involved in the perception and transduction of symbiotic signalling molecules has become increasingly apparent. A Ca^2+^-mediated symbiotic signalling pathway has been shown to be activated by symbiosis-related signals not only in the host plant [6], but also in the rhizobial partner [7-10]. Measurements of intracellular Ca^2+^ concentration by means of recombinant aequorin expression demonstrated a tight regulation of cytosolic Ca^2+^ level in *M. loti* and the occurrence of Ca^2+^-based mechanisms to detect changes in the soil environment [7].

A fine-tuned Ca^2+^ homeostatic machinery underlies the use of Ca^2+^ as intracellular messenger for signal transduction in any biological systems, either eukaryotic or bacterial [11,12]. The complex Ca^2+^ homeostasis toolkit of eukaryotic cells is made of several membrane-located active Ca^2+^ transporters (Ca^2+^ pumps and/or Ca^2+^ exchangers), that mediate the efflux of the ion into the extracellular milieau or its sequestration into different intracellular Ca^2+^ stores [13]. Several intracellular compartments are equipped with a variety of soluble Ca^2+^ buffering proteins, able to bind Ca^2+^ with different affinities and capacities [14]. Recent evidence indicates that Ca^2+^ buffers are likely to play a critical role in Ca^2+^ homeostasis also in bacteria. In the cyanobacterium *Anabaena* sp., the Ca^2+^-binding protein CcbP has been shown to be involved in the regulation of intracellular free Ca^2+^ concentration during heterocyst differentiation [15,16]. Based on nuclear magnetic resonance spectroscopy analysis of the structures of Ca^2+^-free and Ca^2+^-bound CcbP forms, it has been proposed that CcbP may represent a novel class of Ca^2+^-binding proteins, involved in the cytosolic sequestration of the ion in *Anabaena* sp. [17].

In this work, we investigated the possible occurrence of Ca^2+^ binding proteins as a component of the Ca^2+^ homeostatic machinery of the nitrogen-fixing bacterium *M. loti*. Herewith we provide evidence for the presence of a small soluble acidic protein, identified by Q-TOF MS/MS analyses as ferredoxin II that, on the basis of its biochemical features, may act as a major Ca^2+^ buffer in this rhizobial species.

## Results

### Isolation and biochemical characterization of Ca^2+^ buffering proteins in *Mesorhizobium loti*

As a first step in the search for a proteinaceous Ca^2+^-buffering system in *M. loti*, we considered the possibility that rhizobia might express proteins sharing a certain degree of amino acid sequence homology with the Ca^2+^-binding protein CcbP of the cyanobacterium *Anabaena*, which has been shown to regulate the bacterial intracellular free Ca^2+^ concentration during heterocyst differentiation [15-17]. Bioinformatic searches into the complete genome of *M. loti* [3] did not highlight any gene sharing a significant degree of nucleotide sequence identity with the *Anabaena* CcbP-encoding gene (GenBank accession no. AY919604). As a consequence, a biochemical approach was undertaken to search Ca^2+^-binding proteins potentially acting as Ca^2+^ buffers in *M. loti*.

**Figure 1:**
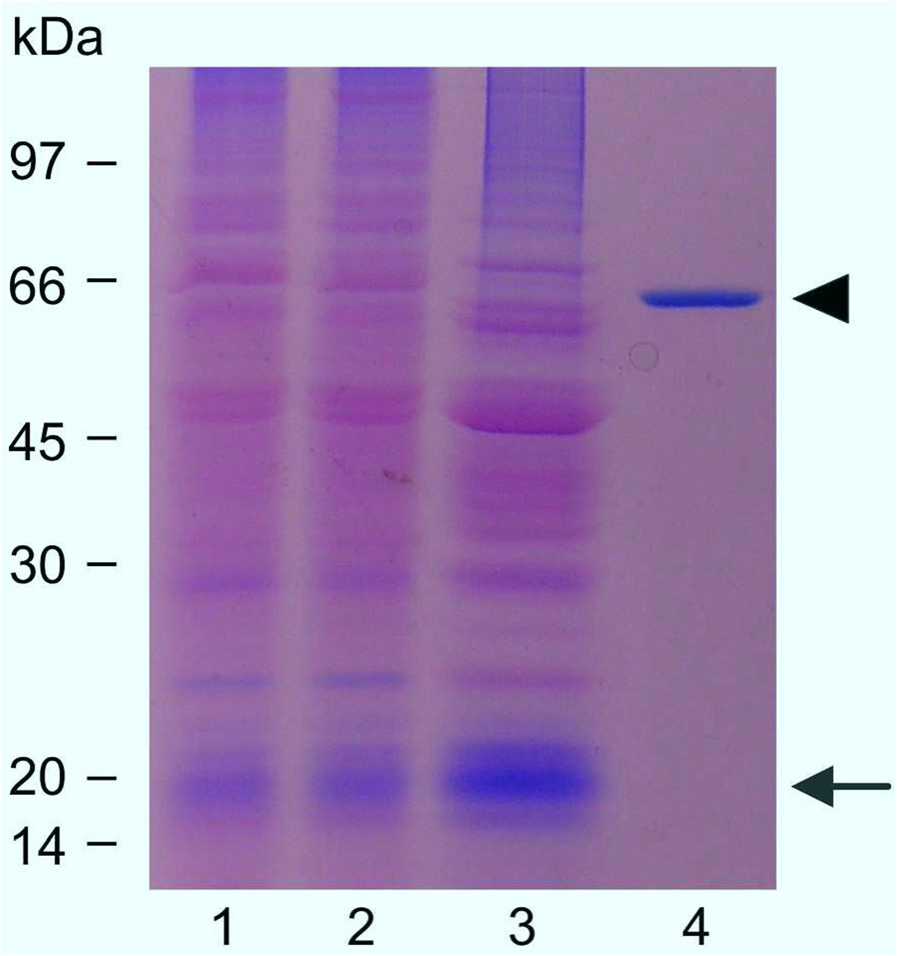
**Isolation of acidic Ca** ^**2+**^ **buffering proteins from** ***M*** ***.*** ***loti*** **.** Stains-all staining of SDS-PAGE (10-12.5% polyacrylamide linear gradient gel). Acidic proteins which bind Ca^2+^, such as calsequestrin and calreticulin, stain blue with Stains-all, whereas other proteins stain pink [21]. Lane 1, *M. loti* crude extracts (50 μg); lane 2, soluble fraction (50 μg); lane 3, protein sample after selective precipitation with ammonium sulphate (50 μg); lane 4, calsequestrin purified from rabbit skeletal muscle, used as a positive control (1 μg). Arrow, *M. loti* Stains-all blue-staining band with an apparent molecular mass of 20 kDa; arrowhead, rabbit calsequestrin.

Crude protein extracts were prepared from mid-exponential phase cultures of *M. loti* strain 3147^T^ and fractionated into a soluble fraction and a membrane fraction. To isolate soluble Ca^2+^ buffering proteins, a selective ammonium sulfate precipitation procedure, followed by anion exchange chromatography on DEAE-Cellulose, was applied. This experimental procedure has been traditionally used for the purification of acidic Ca^2+^-binding proteins, such as calsequestrin [18] and calreticulin from both animal [19] and plant [20] tissues. The same procedure has been later adopted for the isolation of the acidic Ca^2+^-binding protein CcbP from of the cyanobacterium *Anabaena* [15]. After separation on SDS-PAGE of protein samples, the slab gels were incubated with the cationic carbocyanine dye Stains-all, that is commonly used for the preliminary detection of acidic Ca^2+^-binding proteins [21]. As shown in Figure 1, a *M. loti* protein with an apparent molecular mass of about 20 kDa stained blue with Stains-all, whereas most other proteins stained pink. As a positive control, calsequestrin, purified from rabbit skeletal muscle, was used (Figure 1). The soluble *M. loti* Stains-all blue-staining protein was eluted from the DEAE-Cellulose column at 0.45-0.55 M NaCl, showing a several-fold level of enrichment (Additional file 1).

**Figure 2:**
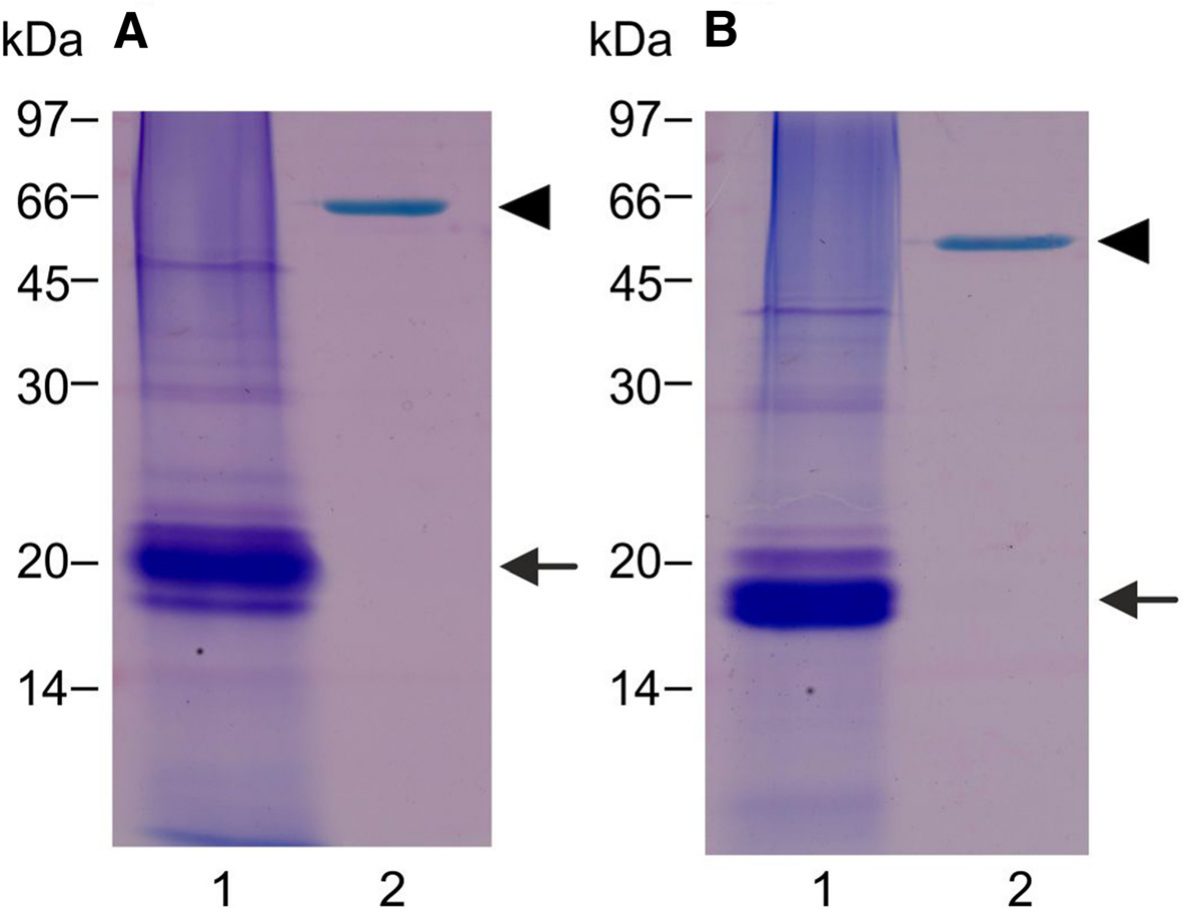
**Ca** ^**2+**^ **-** **dependent changes of electrophoretic mobility.** Proteins were electrophoresed on 12.5% SDS-PAGE either in the presence of 1 mM EGTA **(A)** or 1 mM CaCl_2_ **(B)** and stained with Stains-all. Lane 1, 20 kDa *M. loti* protein partially purified by DEAE-Cellulose (2 μg, arrow); lane 2, rabbit calsequestrin, used as positive control (0.5 μg, arrowhead).

It has to be noted that Stains-all blue-staining cannot be considered as necessarily diagnostic for acidic Ca^2+^-binding proteins, since this dye metachromatically stains also phosphorylated proteins as well as sialoglycoproteins [22,23]. Therefore, the potential ability of the *M. loti* protein to bind Ca^2+^ was further investigated by a Ca^2+^-dependent electrophoretic mobility shift assay [24]. To this aim, *M. loti* proteins partially purified by DEAE-Cellulose were electrophoresed on SDS-PAGE containing either EGTA or CaCl_2_. As shown in Figure 2, the major *M. loti* Stains-all blue-staining band of about 20 kDa shifted to a lower apparent M_r_ in the presence of Ca^2+^, like rabbit calsequestrin did.

**Figure 3:**
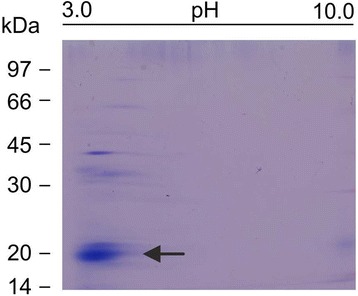
**Isoelectric focusing of the DEAE** **-Cellulose fraction from** ***M*** ***.*** ***loti*** **.** The slab gel was stained with Stains-all. About 15 μg of protein was loaded. Arrow indicates the major Stains-all blue-staining protein band of 20 kDa.

Since eukaryotic high-capacity, low-affinity Ca^2+^ binding proteins such as calsequestrin and calreticulin are characterized by an acidic isoelectric point [25], isoelectric focusing (IEF) analyses of the *M. loti* putative Ca^2+^-binding protein were carried out. The *M. loti* Stains-all blue-staining protein band was indeed found to migrate in the low pH region of the gel (Figure 3) and, when analysed by liquid phase IEF, exhibited a pI of 4.2 (data not shown).

**Figure 4:**
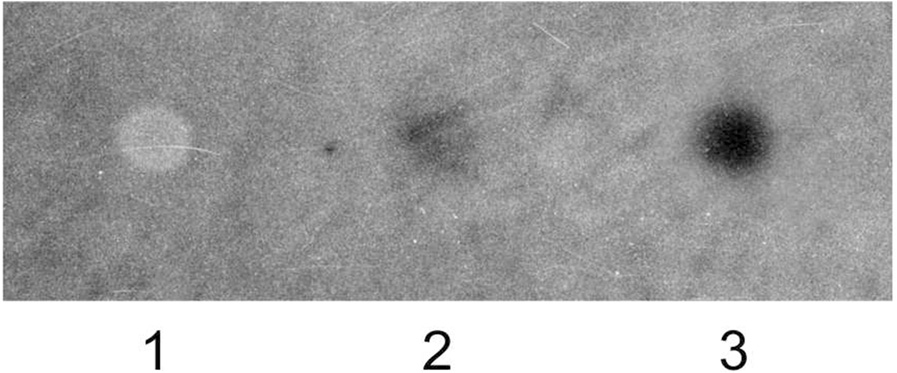
**Identification of** ***M*** ***.*** ***loti*** **Ca** ^**2+**^ **binding proteins by** ^**45**^ **Ca** ^**2+**^ **-ligand overlay.** Proteins were dot-blotted onto nitrocellulose and then incubated with ^45^CaCl_2_ in a calcium overlay assay [44]. ^45^Ca-labeled proteins were visualized by autoradiography (7-d exposure). 1, equine cytochrome c, used as negative control (5 μg); 2, purified *M. loti* 20 kDa Stains-all blue-staining protein (5 μg); 3, rabbit calsequestrin, used as positive control (1 μg).

To further purify the protein to homogeneity, electroendosmotic preparative electrophoresis (EPE) was performed, yielding a fraction containing the virtually pure 20 kDa *M. loti* protein (fraction 8 in Additional file 2). The Ca^2+^ binding ability of the purified protein was confirmed by ^45^Ca^2+^ overlay assays, in which calsequestrin and cytochrome c were used as positive and negative controls, respectively (Figure 4).

### Identification of the *M. loti* Ca^2+^ binding protein by mass spectrometry

**Figure 5:**
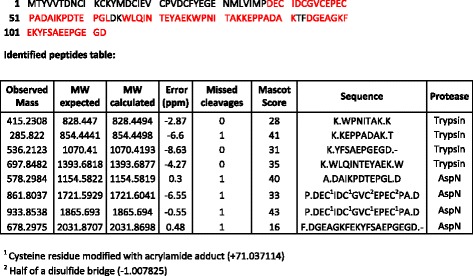
**One letter code amino acid sequence of ferredoxin II** **(mlr3855)** **from** ***Mesorhizobium loti*** **strain MAFF303099.** Peptides identified by MS analysis are colored in red.

**Figure 6:**
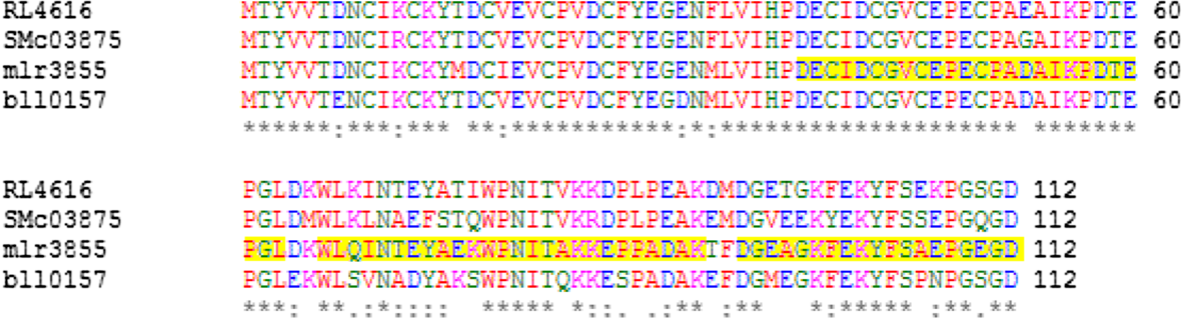
**Alignment of ferredoxin sequences from different rhizobial species.** Ferredoxin II (mlr3855) from *M. loti* MAFF303099 showed 85% amino acid sequence identity with a putative ferredoxin II (RL4616) from *Rhizobium leguminosarum* bv. *viciae* 3841, 75% with a putative ferredoxin protein from *Sinorhizobium meliloti* 1021 (SMc03875) and 83% with ferredoxin (bll0157) from *Bradyrhizobium japonicum* USDA110. mlr3855 sequence covered by MS analysis is highlighted in yellow.

Mass spectrometry (MS) analyses, carried out after digestion with either trypsin or endoproteinase AspN, identified the protein purified from EPE as ferredoxin II. Best coverage and identification results were then obtained from fraction 8, while some ferredoxin II peptides were identified below significance threshold also in fraction 7 and 9 (data not shown). This is probably due to different abundance of the protein in each fraction or to uncovered post-translational modifications of its residues in fraction 7 and 9. Overall sequence coverage of 54% was reached among trypsin and AspN digestion, thus confirming the presence of ferredoxin II in fraction 8 (Figure 5). With reference to the RhizoBase at Kazusa Genome Resources, there are five ferredoxins (msl0793, mll5100, mlr5869, mlr5930, msl8750) plus a ferredoxin-like protein (msl5859) and a probable ferredoxin (msr9193) in *Mesorhizobium loti* with low sequence homology to the one identified by our analyses (mlr3855). Moreover, we retrieved sequences from at least other three ferredoxins spanning high sequence homology to identified mlr3855 in *Rhizobium leguminosarum*, *Sinorhizobium meliloti* and *Bradyrhizobium japonicum*, thus supporting a relevant role for this protein in nitrogen-fixing bacteria (Figure 6). *In silico* sequence analysis with ScanProsite tool [26] (http://prosite.expasy.org/scanprosite/) confirmed the presence of two 4Fe-4S ferredoxin-type iron-sulfur binding domain profiles between residues 1-30 and 31-60 in the cysteine-rich region of mlr3855, which we partially covered by MS analysis.

Interestingly, the doubly charged tryptic fragment YFSAEPGEGD (m/z 536.2123) was also identified in a form modified by an adduct with Ca^2+^ (m/z 555.1855) (Additional file 3), further strengthening the notion that the *M. loti* protein is indeed a Ca^2+^-binding protein.

The protein has a predicted molecular mass of 12553.13 Da and a theoretical pI of 4.18, in perfect agreement with the value (4.2) identified by IEF analyses. The discrepancy between the actual molecular mass and the apparent molecular weight on SDS-PAGE (Figures 1, 2, 3) is a common feature of acidic Ca^2+^ binding proteins, which often migrate anomalously in Laemmli SDS-PAGE [25].

## Discussion

In this work we have provided evidence for the occurrence of a major soluble Ca^2+^ binding protein in the nitrogen-fixing bacterium *M. loti* USDA 3147^T^ strain. This small acidic protein, which was isolated by using an experimental procedure commonly adopted for the extraction and purification of calsequestrin and calreticulin from animal tissue homogenates, was identified as ferredoxin II by tandem mass spectrometry analyses with an ESI-Q-TOF instrument. Ferredoxin II shares with eukaryotic Ca^2+^ buffering proteins several biochemical features, *i.e.* metachromatic staining with Stains-all, an acidic isoelectric point, the Ca^2+^-dependent change of electrophoretic mobility and the Ca^2+^-binding ability in ^45^Ca^2+^ overlay assay. All these characteristics, together with mass spectrometry evidence for a Ca^2+^ adduct in the C-terminal region peptide YFSAEPGEGD, suggest that ferredoxin II may participate in Ca^2+^ homeostasis in *M. loti*.

Further investigations are needed to unravel the physical basis for ferredoxin II ability to bind Ca^2+^, since the protein lacks classical EF-hand motifs. However, it is possible to speculate that the abundance of negatively charged amino acid residues present in the C-terminal region of the protein, where aspartic and glutamic acid residues reach 40% of the sequence along the last ten residues, may play some role, as hypothesized for CP12, a novel Ca^2+^-binding protein which has recently been identified in the chloroplasts of *Arabidopsis thaliana* [27]. Moreover, based on the class assignment for solvent accessibility of the tool NetSurfP - Protein Surface Accessibility and Secondary Structure Prediction (Technical University of Denmark, http://www.cbs.dtu.dk/services/NetSurfP/), the last eight residues encompassing two glutamic and one aspartic acid are exposed to solvent in mlr3855, thus they are possibly accessible to Ca^2+^ and prone to formation of adducts, like the one confirmed here by MS/MS data.

Ca^2+^ is acknowledged as a ubiquitous and versatile intracellular messenger, able to transduce a wide variety of signals into proper cellular responses in all eukaryotic cells [13,28,29]. Increasing evidence suggest that the basic components of Ca^2+^ homeostasis and signalling machineries are present in bacteria as well [30-32]. In rhizobium-legume symbiosis, Ca^2+^ has been demonstrated to be involved in the perception of symbiotic signals not only by the plant host [33] but also by the bacterial partner. In particular, several plant-derived diffusible molecules, such as tetronic acid in *M. loti* [7] and the flavonoid naringenin in *R. leguminosarum* [8,10] were found to trigger transient intracellular Ca^2+^ changes leading to the expression of bacterial nodulation genes. Although the molecular identity of the Ca^2+^-permeable channels involved in the observed Ca^2+^ fluxes remains elusive, in this work another essential component of the Ca^2+^ homeostatic machinery, *i.e.* a protein Ca^2+^ buffer, was identified. It has recently been hypothesized that Ca^2+^ buffering proteins, in addition to quickly bind and thus remove free Ca^2+^ ions that can have toxic effect for the cell, can also play a crucial role in shaping the Ca^2+^ signals [34].

In the cyanobacterium *Anabaena* sp. strain PCC 7120 the Ca^2+^-binding protein CcbP was found to play an important role in the regulation of intracellular free Ca^2+^ concentration during heterocyst differentiation [15]. In particular, the protein was found to be degraded by the serine-type protease HetR, thereby releasing bound Ca^2+^ and significantly contributing to the establishment of higher levels of cytosolic Ca^2+^ in mature heterocysts [16].

In a recent comparative proteomic analysis of *M. loti* MAFF303099, no change in the expression of ferredoxin II was found between the free-living and symbiotic condition [35], in agreement with previous results obtained by transcriptomic analysis [36]. Similarly, a putative ferredoxin II encoding gene (RL4616) in *R. leguminosarum* bv. *viciae* was not among the significantly upregulated genes in bacteroids compared with free-living cells [37]. These data suggest that ferredoxin II may play an essential constitutive function in rhizobia as a part of the bacterial Ca^2+^ homeostat. It remains to be established whether the transition from free-living bacteria to bacteroids may involve changes in intracellular free Ca^2+^ concentration in *M. loti*, thereby possibly involving a post-translational regulatory mechanism for the level of ferredoxin II, similar to that described for cyanobacterial CcbP.

It cannot be ruled out that *M. loti* ferredoxin II may play additional roles within the rhizobial cell. Based on the Rhizobase at Kazusa DNA Research Institute, *M. loti* ferredoxin II falls within the functional category of central intermediary metabolism and nitrogen metabolism [35]. Interestingly, a protein sharing a significantly high homology (83%) with *M. loti* ferredoxin II is found also in *Rhodopseudomonas palustri*s, a metabolically versatile photosynthetic bacterium able to fix nitrogen. A relevant similarity between genes of rhizobia and *Rhodopseudomonas* is not unexpected as they share a close terminal branch in 16S rRNA phylogeny [38], which entails recent separation and consequently high colinearity of their genomes.

Indeed, a commonality among eukaryotic organellar Ca^2+^ buffering proteins is to be multifunctional proteins, carrying out additional important functions not necessarily directly related to Ca^2+^ homeostasis [14]. In particular, the presence of Fe-S clusters renders ferredoxin II particularly suited for electron transfer during redox reactions, similarly to ferredoxin. Interestingly, Ca^2+^ binding to a plant ferredoxin has been hypothesized to affect both the buffering capacity of the chloroplast stroma and the formation of complexes with ferredoxin-dependent oxidoreductases [39].

Our findings support the notion that proteins with the ability to bind Ca^2+^ appeared early in the history of life, possibly contributing to the regulation of intracellular free Ca^2+^ levels and to the consequent evolution of Ca^2+^-based signalling mechanisms [12].

## Conclusions

In this work we demonstrated that ferredoxin II is a major Ca^2+^ binding protein in *M. loti*, potentially acting as a primitive system to control intracellular free Ca^2+^ concentration in this rhizobial species. The elucidation of the mechanisms involved in Ca^2+^ homeostasis in rhizobia may help to better understand the Ca^2+^ signalling events underlying the early stages of nitrogen-fixing symbiosis with host legumes.

## Methods

### Strain and growth conditions

*M. loti* USDA strain 3147^T^, kindly provided by P. Van Berkum (USDA, Beltsville MD), was cultured in minimal BIII medium [40] at 28°C, as previously described [7]. For protein sample preparations, cells were harvested at OD_600nm_ = 0.6 (mid-growth phase).

### Isolation and purification of acidic Ca^2+^ binding proteins

To prepare protein crude extracts from *M. loti* suspension cell cultures, cells were harvested by centrifugation at 3000 *g* for 20 min at 4°C, washed twice with fresh medium, centrifuged again and resuspended in 4 volumes of phosphate-buffered saline (PBS) supplemented with 0.5 mM benzamidine and 0.5 mM phenylmethylsulfonyl fluoride. Bacteria were lysed by 3 cycles of 30 s each of sonication at 35 Hz (Fisher Sonic, Artek Farmingdale, NY, USA), each followed by 30 s on ice. After sonication, samples were centrifuged at 1600 *g* for 15 min at 4°C to pellet down and discard non lysed bacteria.

Total protein extracts (from about 25 g cells, fresh weight) were further fractionated in a soluble and membrane fractions by centrifugation at 150.000 *g* for 45 min at 4°C: the supernatant, containing soluble proteins, was subjected to a selective ammonium sulfate precipitation procedure followed by DEAE-Cellulose column chromatography, as described by Slupsky et al. [18]. Protein fractions from the DEAE-Cellulose column (DE52, Whatman, Maidstone, UK) collected between 0.4 and 0.5 M NaCl were further purified by using electroendosmotic preparative electrophoresis (EPE) [41], which was carried out with an ELFE apparatus (Genenco Life Science, M-Medical srl, Firenze, Italy). Protein concentration was determined by using the Bio-Rad Protein Assay (Bio-Rad Laboratories, Hercules, CA, USA), according to manufacturer’s instructions.

In some experiments calsequestrin, purified from rabbit skeletal muscle as previously described [42] was used as positive control.

### Gel electrophoresis analyses

One-dimensional SDS-PAGE was performed according to Laemmli [43], using 10 to 12.5% polyacrylamide linear gradient gels or 12.5% gels. Ca^2+^-dependent electrophoretic mobility shift assays were performed by separating protein samples on SDS-PAGE slab gels containing either 1 mM EGTA or 1 mM CaCl_2_, both in the stacking and in the separating gels [24].

For in gel-isoelectric focusing (IEF), purified *M. loti* proteins were thoroughly desalted and resuspended in 0.2 ml of 9 M urea, 2 M thiourea, 4% (w/v) 3-[(3-cholamidopropyl) dimethylammonio]-1-propanesulfonate (CHAPS) and 2 mM tris(2-carboxyethyl)phosphine (TCEP) using Micro Bio-Spin columns (Bio-Rad). Ampholytes, 3-10 pH range (Bio-Rad), and bromophenol blue were then added to the sample at 0.2% and 0.01% (w/v) final concentration, respectively. Isoelectric focusing (IEF) was performed on 11 cm IEF strips, 3-10 pH range (Bio-Rad). When 7 cm IEF strips, pH range 4-7 (Bio-Rad), were used, the separation was performed on 0.125 ml sample, in the presence of ampholytes, 4-7 pH range (Bio-Rad), (0.2%, w/v), for 10,000 V/h. Before the second dimension separation on a 10-12.5% gradient SDS-PAGE, the strips were equilibrated in 10 ml of 0.375 M tris-HCl, pH 8.8 containing 2% (w/v) SDS, 6 M urea, 20% (v/v) glycerol and 2% DTT for 10 minutes and then in the same buffer with 2.5% (w/v) iodoacetamide.

Liquid-phase IEF was instead performed by using a MicroRotofor Cell (Bio-Rad). In this case *M. loti* proteins were thoroughly desalted on NAP 5 columns (GE Healthcare, Little Chalfont, Buckinghamshire, UK) and resuspended in 2.5 ml of focusing buffer: 7 M urea, 2 M thiourea, 4% (w/v) CHAPS, 2 mM TCEP and 3% (w/v) ampholytes, 3-10 pH range. Proteins were separated at constant power (1 W) for 5 h using 0.1 M H_3_PO_4_ at the anode and 0.1 M NaOH at the catode. At the end of the run ten protein fractions of almost the same volume were collected and used for further studies.

Slab gels were stained with Coomassie Blue, completely destained and then restained with Stains-all, as described by Campbell et al. [21].

### ^45^Ca^2+^ overlay assay

^45^Ca^2+^ overlay assay was carried out by the technique described by Maruyama et al. [44]. Proteins were spotted directly onto nitrocellulose membranes. Rabbit calsequestrin was used as positive control, equine cytochrome c (Sigma-Aldrich) was used as negative control. ^45^CaCl_2_ was purchased from Perkin-Elmer (Boston, MA, USA). ^45^Ca^2+^-labeled proteins were visualized by autoradiography on Amersham Hyperfilm MP (GE Healthcare).

### Q-TOF analyses

For Q-TOF analyses, proteins purified by the EPE technique (fractions 7-8-9 separately) were used, after buffer exchange with Vivaspin 6 concentrators with a 5000 MW cut-off (Sartorius Stedim Biotech GmbH, Goettingen, Germany) against 40 mM NH_4_HCO_3_, 10% (v/v) acetonitrile pH 8.0. Each fraction was then digested either with trypsin and endoproteinase AspN based on obtainable coverage predicted *in silico* [45] (http://web.expasy.org/peptide_mass/). 100 ng of each protease were used for overnight digestion at 37°C and reaction was stopped by acidification with formic acid (FA) 1% (v/v) final concentration. Digests were dried by vacuum centrifugation and resuspendend into a minimal volume of 0.1% FA for subsequent MS analysis. 1 to 10 μl of each peptide mixture, obtained from the digestion of protein samples, were analyzed by means of reversed-phase chromatography on a nano-fluidic HPLC-Chip apparatus coupled with a quadrupole ion trap and time of flight mass spectrometer, using the 6520 Accurate-Mass Q-TOF LC/MS system (Agilent Technologies) equipped with MassHunter Workstation Software Qualitative Analysis B.02.00 as graphical interface for data handling. A 1200 Rapid Resolution system (Agilent Technologies, Santa Clara, CA, USA) containing a binary pump and degasser and a well-plate autosampler with thermostat were associated to the HPLC-Chip interface directly connected to a nanoESI ionization source. Loaded samples were thus enriched on a 160 nl enrichment column and separated with an acetonitrile gradient on a 75 μm x 150 mm separation column packed with Zorbax 300SB-C18 5 μm material. Data dependent MS/MS analysis were carried on the 3th most intense peaks from each MS scan using collision induced dissociation fragmentation (CID) with dynamic exclusion in order to enhance sequencing of less abundant peptides.

### Data analysis

MS/MS data were extracted for identification purpose from raw data by means of MassHunter Workstation Software Qualitative Analysis and converted to Mascot generic format datafile (.mgf). Thus, data were analysed with Mascot server (ver. 2.3, Matrix Science Ltd.) being classified by a probability based implementation of the Mowse algorithm: experimental mass spectra produced were correlated to peptide sequences obtained by comparison with the theoretical mass spectra in the RhizoBase protein database downloaded from the Kazusa DNA Research Institute website (http://genome.microbedb.jp/RhizoBase). As the confidence of protein identification a precursor mass tolerance of no more than 10 ppm and MS/MS product mass tolerance of no more than 0.05 Da were adopted. Methionine oxidation, asparagine/glutamine deamidation and cysteine disulfide or acrylamide adducts formation were considered as routine possible post-translational modification (PTM). Sequence alignments were performed by means of ClustalW2 alignment tool (http://www.ebi.ac.uk/Tools/msa/clustalw2/) using Gonnet protein weight matrix.

### Accession numbers

All MS/MS data were searched with Mascot server 2.3 against *Mesorhizobium loti* protein sequences downloaded from the RhizoBase database (http://genome.microbedb.jp/rhizobase/Mesorhizobium/genes.faa). Accession numbers of identified proteins refers to the ones contained in this database.

## Additional files

Additional file 1:
**Purification of** ***M. loti*** **acidic Ca** ^**2+**^ **buffering proteins by DEAE-Cellulose chromatography.** **A**: Elution profile of *M. loti* proteins on DEAE-Cellulose chromatography. The column was eluted with a 50 mM - 1 M NaCl linear gradient, as indicated. **B**: Stains-all staining of 10-12.5% SDS-PAGE. Key to lanes: L, protein mixture loaded onto the column, after selective precipitation with ammonium sulphate (10 μg); V, void volume (10 μg); 1-12, fractions eluted from the DEAE-Cellulose column (100 μl each, containing 1-10 μg protein). The protein fraction size was 2 ml. Additional file 2:
**SDS-PAGE analysis of samples obtained from the fractionation of** ***M. loti*** **proteins by electroendosmotic preparative electrophoresis.** Protein fractions (fraction size: 1 ml) were electrophoresed on 12.5% SDS-PAGE (18 μl per lane) and stained with Stains-all. Only fractions 1 to 9 are shown. Molecular masses of standard proteins are indicated on the left side of the gel. Additional file 3:
**YFSAEPGEGD mlr3855 peptide identification data with and without Ca** ^**2+**^ **adduct on glutamic acid residue.** **A**: MS/MS spectra (left) and matched MS/MS fragment masses (right) for peptide YFSAEPGEGD at m/z 536.2123, 2+ at retention time 11.124 minutes. **B**: MS/MS spectra (left) and matched MS/MS fragment masses (right) for peptide YFSAEPGEGD modified with Ca^2+^ cation adduct on glutamic acid residue in position #5 at m/z 555.1855, 2+ at retention time 11.039 minutes. b and y refers to CID fragmentation ionic series, b/y^0^ are –H_2_O fragment ions and b/y^++^ are doubly charged fragment ions according to Mascot nomenclature (Matrix Sciences).
