# Supplementary figures and images for: Identification of ferredoxin II as a major calcium binding protein in the nitrogen-fixing symbiotic bacterium Mesorhizobium loti
Source: BMC Microbiol. 2015 Feb 4;15(1):16. doi: 10.1186/s12866-015-0352-5 (PMC4322793; doi:10.1186/s12866-015-0352-5)

**A**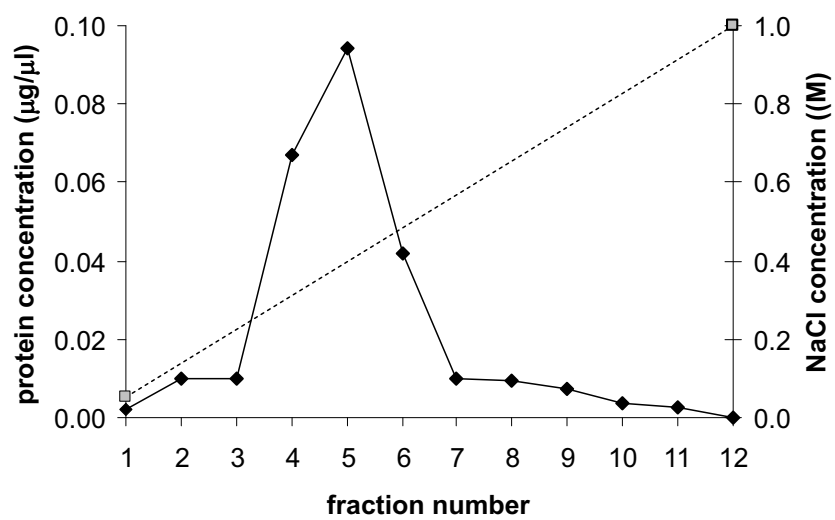**B**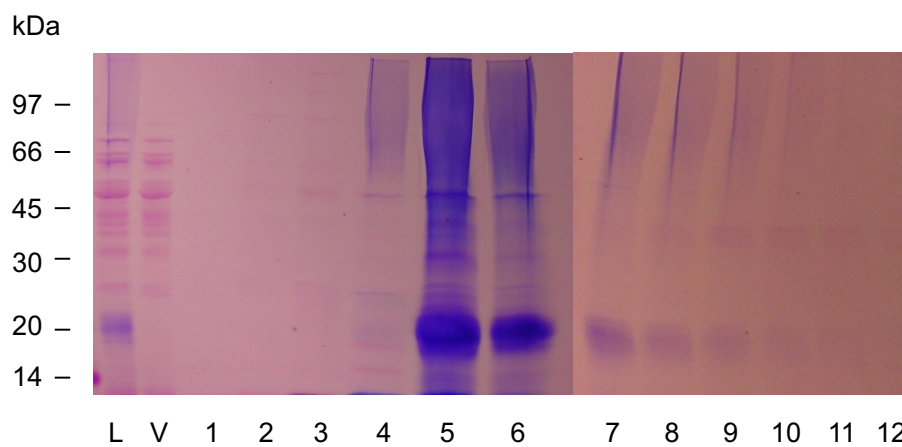

Supplement: Additional file 1: — Purification of M. loti acidic Ca 2+ buffering proteins by DEAE-Cellulose chromatography. A: Elution profile of M. loti proteins on DEAE-Cellulose chromatography. The column was eluted with a 50 mM - 1 M NaCl linear gradient, as indicated. B: Stains-all staining of 10-12.5% SDS-PAGE. Key to lanes: L, protein mixture loaded onto the column, after selective precipitation with ammonium sulphate (10 μg); V, void volume (10 μg); 1-12, fractions eluted from the DEAE-Cellulose column (100 μl each, containing 1-10 μg protein). The protein fraction size was 2 ml. [file 12866_2015_352_MOESM1_ESM.pdf]

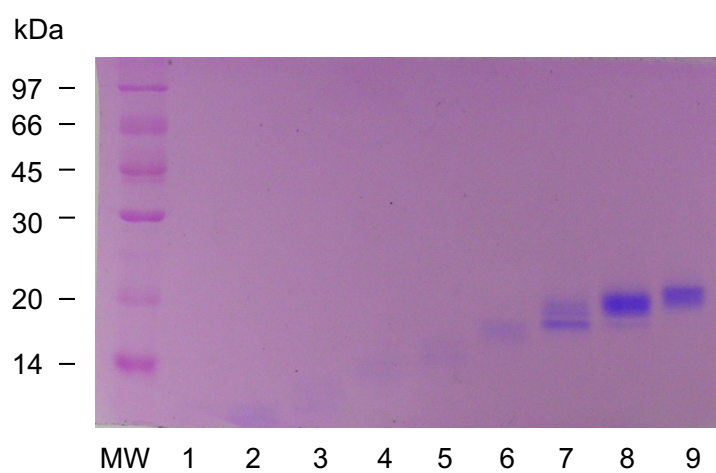

Supplement: Additional file 2: — SDS-PAGE analysis of samples obtained from the fractionation of M. loti proteins by electroendosmotic preparative electrophoresis. Protein fractions (fraction size: 1 ml) were electrophoresed on 12.5% SDS-PAGE (18 μl per lane) and stained with Stains-all. Only fractions 1 to 9 are shown. Molecular masses of standard proteins are indicated on the left side of the gel. [file 12866_2015_352_MOESM2_ESM.pdf]
