# Supplementary material for: Identification of ferredoxin II as a major calcium binding protein in the nitrogen-fixing symbiotic bacterium Mesorhizobium loti
Source: BMC Microbiol. 2015 Feb 4;15(1):16. doi: 10.1186/s12866-015-0352-5 (PMC4322793; doi:10.1186/s12866-015-0352-5)
Supplement: Additional file 3: — YFSAEPGEGD mlr3855 peptide identification data with and without Ca 2+ adduct on glutamic acid residue. A: MS/MS spectra (left) and matched MS/MS fragment masses (right) for peptide YFSAEPGEGD at m/z 536.2123, 2+ at retention time 11.124 minutes. B: MS/MS spectra (left) and matched MS/MS fragment masses (right) for peptide YFSAEPGEGD modified with Ca2+ cation adduct on glutamic acid residue in position #5 at m/z 555.1855, 2+ at retention time 11.039 minutes. b and y refers to CID fragmentation ionic series, b/y0 are –H2O fragment ions and b/y++ are doubly charged fragment ions according to Mascot nomenclature (Matrix Sciences). [file 12866_2015_352_MOESM3_ESM.pdf]

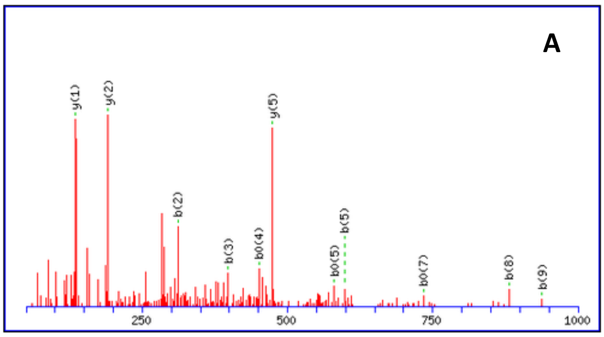

#	b	b^{++}	b^0	b^{0++}	Seq.	y	y^{++}	y^0	y^{0++}	#
1	164.0706	82.5389			Y					10
2	*311.1390*	156.0731			F	908.3632	454.6852	890.3527	445.6800	9
3	*398.1710*	199.5892	380.1605	190.5839	S	761.2948	381.1510	743.2842	372.1458	8
4	469.2082	235.1077	451.1976	226.1024	A	674.2628	337.6350	656.2522	328.6297	7
5	*598.2508*	299.6290	580.2402	290.6237	E	603.2257	302.1165	585.2151	293.1112	6
6	695.3035	348.1554	677.2930	339.1501	P	**474.1831**	237.5952	456.1725	228.5899	5
7	752.3250	376.6661	734.3144	367.6608	G	377.1303	189.0688	359.1197	180.0635	4
8	*881.3676*	441.1874	863.3570	432.1821	E	320.1088	160.5581	302.0983	151.5528	3
9	*938.3890*	469.6982	920.3785	460.6929	G	**191.0662**	96.0368	173.0557	87.0315	2
10					D	**134.0448**	67.5260	116.0342	58.5207	1

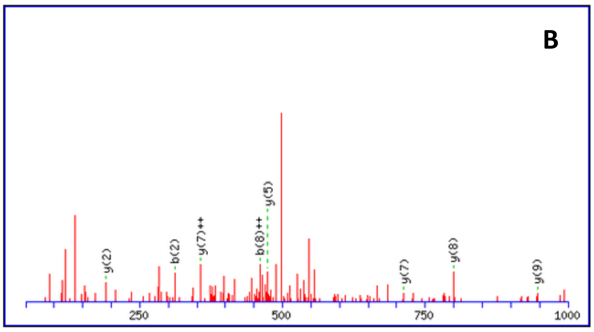

#	b	b^{++}	b^0	b^{0++}	Seq.	y	y^{++}	y^0	y^{0++}	#
1	164.0706	82.5389			Y					10
2	**311.1390**	156.0731			F	*946.3102*	473.6587	928.2996	464.6534	9
3	398.1710	199.5892	380.1605	190.5839	S	*799.2417*	400.1245	781.2312	391.1192	8
4	469.2082	235.1077	451.1976	226.1024	A	*712.2097*	356.6085	694.1992	347.6032	7
5	636.1977	318.6025	618.1871	309.5972	E	641.1726	321.0899	623.1620	312.0847	6
6	733.2505	367.1289	715.2399	358.1236	P	*474.1831*	237.5952	456.1725	228.5899	5
7	790.2719	395.6396	772.2614	386.6343	G	377.1303	189.0688	359.1197	180.0635	4
8	919.3145	**460.1609**	901.3040	451.1556	E	320.1088	160.5581	302.0983	151.5528	3
9	976.3360	488.6716	958.3254	479.6663	G	*191.0662*	96.0368	173.0557	87.0315	2
10					D	134.0448	67.5260	116.0342	58.5207	1
